# The Effect of N-Acetylcysteine on Respiratory Enzymes, ADP/ATP Ratio, Glutathione Metabolism, and Nitrosative Stress in the Salivary Gland Mitochondria of Insulin Resistant Rats

**DOI:** 10.3390/nu12020458

**Published:** 2020-02-12

**Authors:** Anna Zalewska, Izabela Szarmach, Małgorzata Żendzian-Piotrowska, Mateusz Maciejczyk

**Affiliations:** 1 Experimental Dentistry Laboratory, Medical University, 15-222 Bialystok, Poland; 2 Department of Orthodontics, Medical University, 15-222 Bialystok, Poland; orthod@umb.edu.pl; 3 Department of Hygiene, Epidemiology and Ergonomics, Medical University, 15-222 Bialystok, Poland; mzpiotrowska@gmail.com

**Keywords:** apoptosis, inflammation, insulin resistance, NAC, salivary glands, mitochondrial activity, nitrosative stress

## Abstract

This is the first study to assess the effect of N-acetylcysteine (NAC) on the mitochondrial respiratory system, as well as free radical production, glutathione metabolism, nitrosative stress, and apoptosis in the salivary gland mitochondria of rats with high-fat diet (HFD)-induced insulin resistance (IR). The study was conducted on male Wistar rats divided into four groups of 10 animals each: C (control, rats fed a standard diet containing 10.3% fat), C + NAC (rats fed a standard diet, receiving NAC intragastrically), HFD (rats fed a high-fat diet containing 59.8% fat), and HFD + NAC (rats fed HFD diet, receiving NAC intragastrically). We confirmed that 8 weeks of HFD induces systemic IR as well as disturbances in mitochondrial complexes of the parotid and submandibular glands of rats. NAC supplementation leads to a significant increase in the activity of complex I, II + III and cytochrome c oxidase (COX), and also reduces the ADP/ATP ratio compared to HFD rats. Furthermore, NAC reduces the hydrogen peroxide production/activity of pro-oxidant enzymes, increases the pool of mitochondrial glutathione, and prevents cytokine formation, apoptosis, and nitrosative damage to the mitochondria in both aforementioned salivary glands of HFD rats. To sum up, NAC supplementation enhances energy metabolism in the salivary glands of IR rats, and prevents inflammation, apoptosis, and nitrosative stress.

## 1. Introduction

N-acetyl-cysteine (NAC) is a derivative of a thiol-containing amino acid, which—directly or indirectly, by increasing the concentration of glutathione—demonstrates antioxidant properties. NAC is a cysteine donor for the synthesis of reduced glutathione (GSH). GSH is the most important intracellular antioxidant maintaining a reduced state of protein thiol groups, which is a prerequisite for sustaining the activity of these proteins. GSH can directly deactivate oxygen free radicals (ROS) or be used by glutathione peroxidase as a cofactor in the detoxification of hydrogen peroxide and peroxynitrite. NAC also stimulates the activity of glutathione reductase (GR), an enzyme that restores the reduced form of glutathione, using NADPH [1].

It has been evidenced that NAC reacts rapidly with highly reactive oxygen and nitrogen radicals. In pH 7 and at room temperature, the rate of HO^•^ elimination is (1.36 × 10^10^ M^−1^.s^−1^), the rate of NO_2_^•^ nitrogen dioxide elimination is (1.0 × 10^7^ M^−1^.s^−1^), and the rate for carbonate radical CO_3_^•−^ is (1.0 × 10^7^ M^−1^.s^−1^). Thus, NAC neutralizes free radicals produced by activated leukocytes, and is able to chelate transition metal ions, such as Cu^2+^ and Fe^3+^ as well as heavy metal ions, such as Cd^2+^, Hg^2+^, and Pb^2+^ by creating complexes with thiols, that are easily removable from the body [2]. Chelation of Cu and Fe ions prevents Fenton reactions and thus the production of one of the most dangerous free radicals: The hydroxyl radical. NAC inhibits the activation of nuclear factor kappa B and reduces subsequent expression of inflammatory cytokines [3]. It also lowers the degree of lipid peroxidation as well as the generation of superoxide anions by activating polymorphonuclear leukocytes independently of calcium [4]. Moreover, studies have demonstrated that NAC may affect the respiratory chain in the mitochondria, and inhibit the apoptotic pathway [5]. Experimental studies have indicated that long-term administration of NAC improves heart and brain mitochondrial activity [6] and prevents senile weakening of complexes I, IV, and V in hepatocyte mitochondria [7]. In vitro studies have shown that NAC supplementation has a protective effect on cytochrome c oxidase and complexes I, IV, and V, prevents drops in ATP concentration [8,9] and protects mitochondrial potential [10]. It was also demonstrated that NAC restores mitochondrial transport and improves calcium uptake in the cells of injured rat brains [11], which was attributed to the redox state of thiol groups in mitochondrial complexes.

It should be emphasized that excessive production of ROS, accompanied by a shortage of antioxidants, results in a condition defined as oxidative stress (OS). Oxidative stress leads to disturbances of cell metabolism and degradation of all cell components [12], resulting in cell death and dysfunctions of organs. Therefore, in recent years we have observed a growing interest in the therapeutic effect of NAC in a wide range of diseases in which OS plays a key role in initiation as well as progression.

In our previous studies we demonstrated that insulin resistance (IR) induced by a high-fat diet interferes with antioxidant systems of parotid and submandibular salivary glands, leading to the oxidation of DNA, proteins, and lipids. This was associated with morphological changes of salivary gland parenchyma, and it disturbed the qualitative and quantitative composition of saliva [13,14,15]. We also observed that IR induced by eight-week high-fat feeding impairs mitochondrial function in both salivary glands by enhancing ROS production and apoptosis, and demonstrated that the mitochondrial system and NOX both increase oxidative and nitrosative stress in both salivary glands [16]. We proved that NAC supplementation strengthens both enzymatic and non-enzymatic antioxidant mechanisms of parotid as well as submandibular glands, and prevents oxidative damage to and dysfunction of the parotid gland of rats with IR induced by high-fat diet [15]. Salivary redox imbalance increases oral cavity diseases associated with OS (tooth decay, gingivitis, periodontitis, oral mucosa ulceration, candidiasis, or burning mouth syndrome) [17,18,19,20]. Indeed, such lesions are found in a large percentage of people with obesity, insulin resistance, or diabetes mellitus [17,21,22,23,24]. Therefore, it is essential to maintain/restore effective functioning of antioxidant systems and thus maintain ROS concentration at a level enabling salivary redox balance.

However, so far there have been no studies evaluating the effect of NAC on mitochondrial oxidative/nitrosative stress and apoptosis, and no studies have assessed whether or not this substance can rescue mitochondrial function in the salivary glands in the rat model of IR.

Therefore, the aim of our study was to examine the effect of NAC supplementation on salivary glands: Their mitochondrial respiratory system and function, mitochondrial ROS production and glutathione metabolism, the activity of NOX and XO, as well as some parameters of nitrosative stress and apoptosis in the rat model of insulin resistance.

## 2. Materials and Methods

The study was approved by the Local Ethical Committee for Animal Experiments in Bialystok, No. 21/2017.

### 2.1. Animals

The experiment was performed on male Wistar rats with the initial body weight of approximately 50–72 g. Throughout the entire study, the rats were housed under standard laboratory animal housing conditions (21 °C, 12 h light/dark cycle), with free access to water and food [25].

After six days of adaptation to the conditions in the animal house, the rats were divided into four groups of 10 individuals each:

Group I—(C) control; rats receiving standard type LSM feed (Agropol Motycz Polska) containing 10.3% fat, 24.2% proteins and 65.5% carbohydrates, and 2 mL/kg body weight saline solution intragastrically (once a day, every day for eight weeks);

Group II—(C + NAC) rats receiving standard type LSM feed as well as N-acetylcysteine solution at a dose of 500 mg/kg body weight intragastrically (once a day, every day for eight weeks) at a volume of 2 mL/kg body weight (NAC, Sigma A9165) [26];

Group III—(HFD) rats fed high-fat diet (Research Diet, USA, catalog number D12492) containing 59.8% fat, 20.1% proteins, 20.1% carbohydrates [14] and 2 mL/kg body weight saline solution intragastrically (once a day, every day for eight weeks);

Group IV—(HFD + NAC) rats fed the abovementioned high-fat diet and, intragastrically (once a day, every day for eight weeks), N-acetylcysteine solution at a dose of 500 mg/kg body weight, at a volume of 2 mL/kg body weight.

Intragastric administration of saline and NAC solutions was performed at a fixed time during the day (between 8 and 9 a.m.) by two trained and authorized experts (A.Z., M.M.). Food intake was monitored once a week, and body weight was measured every two days, which allowed us to decide on an appropriate dose of NAC.

After eight weeks, upon an all-night refraining from any food intake, the concentration of glucose was determined, in phenobarbital anesthetic (80 mg × kg^−1^, intraperitoneally), in blood collected from the caudal vein using a glucometer [27]. Then the rates of non-stimulated and stimulated saliva secretion were measured (pilocarpine hydrochloride at a dose of 5 mg/kg body weight, intraperitoneally; Sigma, Chemical Co., St Louis, MO, USA) in rats of all groups [15].

Next, blood was collected from the abdominal artery, and submandibular and parotid salivary glands were taken. The tissues were freeze-clamped with aluminum tongs, precooled in liquid nitrogen, and stored at −80 °C until the redox assays (but not longer than four weeks).

The plasma insulin level was determined by a commercial ELISA kit (Shibayagi Co., Gunma, Japan) according to the manufacturer’s instructions. To confirm IR, insulin sensitivity was calculated by HOMA-IR index (homeostasis model assessment of insulin resistance = fasting insulin [U/mL] × fasting glucose [mM]/22.5) [28]. Plasma free fatty acids (FFA) were analyzed by gas chromatography (GC) [29].

### 2.2. Mitochondria Isolation

On the day of the biochemical part of the experiment, the collected salivary glands were slowly thawed at 4 °C, weighed and cut into small pieces with scissors. Thus prepared, glands were homogenized (1:10, *w/v*) with a Teflon-and-glass electric homogenizer in ice-cold mitochondrial isolation buffer containing 250 mM sucrose, 5 mM Tris-HCl and 2 mM ethylene glycol bis(2-aminoethyl) tetraacetic acid (EGTA), pH 7.4. To prevent protein degradation and proteolysis, protease inhibitors were added. Homogenate was centrifuged (500× *g*, 10 min, 4 °C) and the resulting supernatant was centrifuged twice at 8000× *g* for 10 min at 4 °C. The mitochondria pellet was resuspended in isolation buffer and used on the same day for assays.

In the mitochondrial fraction we did not detect cytoplasmic glyceraldehyde 3-phosphate dehydrogenase or the nuclear marker histone H3 (data not shown, western blot technique), which excludes extra-mitochondrial changes and proves purity of this fraction [30].

### 2.3. Mitochondrial Assays

The following were assayed in the mitochondrial fraction: The activity of mitochondrial complexes, ADP/ATP ratio, rate of hydrogen peroxide production, glutathione metabolism, pro-oxidative enzymes, nitrosative stress, and selected markers of inflammation and apoptosis. All the determinations were performed in duplicate samples and the results were standardized to 1 mg of total protein. The total protein content was measured using the bicinchoninic acid (BCA) method. A commercial kit (Thermo Scientific PIERCE BCA Protein Assay; Rockford, IL, USA) was used according to the manufacturer’s instructions. The absorbance/fluorescence/chemiluminescence was measured with Infinite M200 PRO Multimode Microplate Reader from Tecan.

### 2.4. Activity of Mitochondrial Complexes

The activity of complex I (E.C. 1.6.5.3) was measured colorimetrically based on 2,6-dichloroindophenol reduction by electrons accepted from coenzyme Q_1_, reduced after oxidation of NADH (reduced form of nicotinamide adenine dinucleotide) by complex I [31].

The activity of complex II (E.C. 1.3.5.1) and complex II + III (E.C. 1.10.2.2) was measured according to Rustin et al. [32]. The activity of succinate-ubiquinone reductase and succinate-cytochrome c reductase (respectively) were measured colorimetrically.

Cytochrome c oxidase (COX) activity was examined colorimetrically by measuring the oxidation of reduced cytochrome c at 550 nm wavelength [33].

### 2.5. ADP/ATP Ratio and Hydrogen Peroxide Production

The ADP/ATP ratio was measured using a bioluminescent method based on the conversion of ATP by luciferase. A commercial kit (ADP/ATP Ratio Assay Kit ab65313, Abcam, Burlingame, CA, USA) was used according to the manufacturer’s instructions.

Hydrogen peroxide (H_2_O_2_) production was analyzed by measuring the increase in fluorescence at 530/590 nm wavelength due to the reaction of Amplex Red with H_2_O_2_ in the presence of horseradish peroxidase [34]. H_2_O_2_ formation rate was calculated using a standard curve of H_2_O_2_ stabilized solution.

### 2.6. Activity of Citrate Synthase

Citrate synthase (CS) activity was measured colorimetrically using the reaction with 5-thio-2-nitrobenzoic acid generated from 5,5′-dithiobis-2-nitrobenzoic acid during CS synthesis [35].

### 2.7. Reduced and Oxidized Glutathione and Redox Status

The level of total glutathione was measured based on an enzymatic reaction with 5,5′-dithiobis-(2-nitrobenzoic acid) (DTNB), NADPH and glutathione reductase (GR) [36].

Oxidized glutathione (GSSG) was estimated similarly to the assay performed for total glutathione; however, prior to the determination, the samples had been thawed and neutralized to pH 6–7 using 1 M chlorhydrol triethanolamine. Then, samples were incubated with 2-vinylpyridine [36].

The level of reduced glutathione (GSH) was calculated from the difference between the level of total glutathione and GSSG [36].

Redox (oxidation/reduction) status was calculated using the formula: [GSH]^2^/[GSSG].

### 2.8. Pro-Oxidant Enzymes

NADPH oxidase (NOX, E.C. 1.6.3.1) activity was measured by luminescence assay using lucigenin as an electron acceptor [37]. One unit of NOX activity was defined as the quantity of the enzyme required to release 1 nmol of superoxide radical for 1 min.

Xanthine oxidase (XO, E.C. 1.17.3.2.) activity was assessed based on uric acid production by measuring the increase in its optical density at 290 nm wavelength [38]. One unit of XO activity was defined as the amount of the enzyme required to release 1 μmol of uric acid for 1 min.

### 2.9. Inflammation and Apoptosis

Interleukin-1β (IL-1β) level was measured using the enzyme-linked immunosorbent assay (ELISA) method. A commercial kit (IL-1β ELISA kit, R&D Systems; Canada, Minneapolis, MN, USA) was used according to the manufacturer’s instructions.

Caspase-3 (CAS-3, EC 3.4.22.56) activity was measured colorimetrically using Ac-Asp-Glu-Val-Asp-p-nitroanilide as a substrate [39]. The amount of p-nitroaniline released by CAS-3 was measured at 405 nm wavelength.

### 2.10. Nitrosative Stress

Nitric oxide (NO) level was measured colorimetrically using sulfanilamide and N-(1-naphthyl)-ethylenediamine dihydrochloride. The absorbance of the obtained product was measured at 490 nm wavelength [40].

Peroxynitrite level was measured colorimetrically based on peroxynitrite-mediated nitration leading to the formation of nitrophenol [41]. The absorbance of the obtained complex was measured at 320 nm wavelength.

Nitrotyrosine level was measured using the ELISA method. A commercial kit (Nitrotyrosine ELISA; Immundiagnostik AG, Bensheim, Germany) was used according to the manufacturer’s instructions.

### 2.11. Statistical Analysis

The statistical analysis was performed using GraphPad Prism 8 for MacOS (GraphPad Software, La Jolla, CA, USA). Normality of the results distribution was determined using the Shapiro-Wilk test. The results were expressed as mean ± SD. One-way ANOVA, Tukey’s multiple comparisons test, and Pearson correlation coefficient were used. Multiplicity adjusted *p* value was calculated. The statistical significance was defined as *p* < 0.05.

## 3. Results

### 3.1. General Characteristics

Eight weeks of high-fat feeding resulted in a significant increase in rat weight compared to the control rats fed normal chow (*p* < 0.0001). NAC administration prevented rat weight gain as body weight of HFD + NAC rats was significantly lower than of rats fed only high-fat diet (*p* < 0.0001). An eight-week high-fat diet resulted in a considerable increase in the level of glucose (*p* < 0.0001 and *p* < 0.0001, respectively), insulin (*p* < 0.0001 and *p* < 0.0001, respectively) and fatty acids (*p* < 0.0001 and *p* < 0.0001, respectively) compared to both the group fed standard diet and rats fed HFD + NAC solution. The HOMA-IR insulin sensitivity index was significantly higher in the high-fat diet group compared to the controls and rats fed HFD + NAC (*p* = 0.0001, *p* = 0.0001, respectively). The comparison of the described parameters between the control group and HFD + NAC group did not reveal any significant differences. Rats from HFD (*p* = 0.0026) and HFD + NAC (*p* = 0.0083) groups consumed considerably less feed compared to the control rats, with energy efficiency significantly higher in both groups fed high-fat diet (HFD, HFD + NAC) in relation to the controls (*p* = 0.01 and *p* = 0.01, respectively) (Table 1).

The secretion of non-stimulated saliva did not differ significantly between the studied groups, with HFD rats characterized by clearly decreased secretion of stimulated saliva compared to the control group (*p* < 0.0001). NAC supplementation prevented the reduction of stimulated saliva secretion in rats fed high-fat diet, and in this group the stimulated saliva secretion was significantly higher compared to HFD group (*p* < 0.0001) (Table 1).

### 3.2. Activity of Mitochondrial Complexes

**Figure 1:**
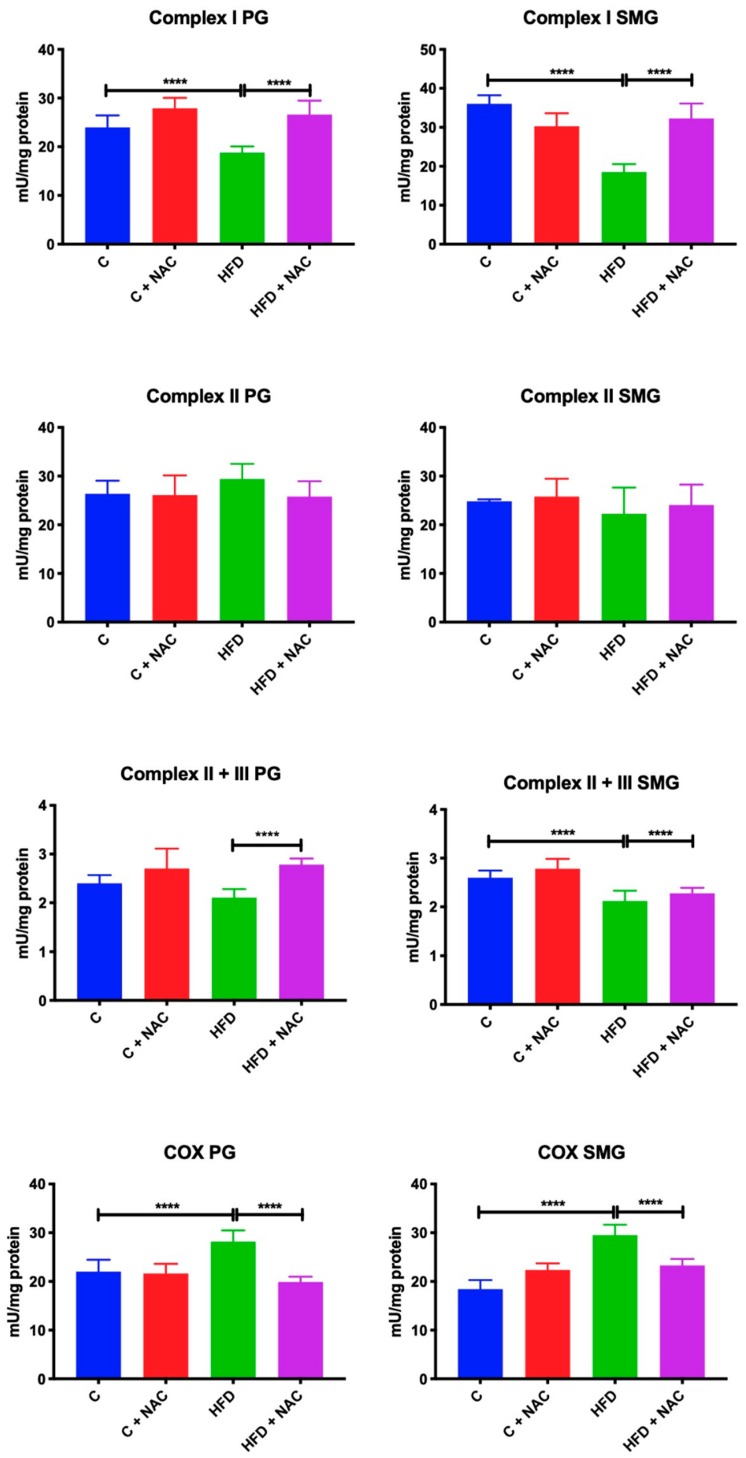
Effect of NAC supplementation on mitochondrial respiratory complexes in the parotid and submandibular glands of insulin resistant rats. C—control rats; C + NAC—control rats + N-acetylcysteine; HFD—rats fed high-fat diet; HFD + NAC—rats fed high-fat diet + N-acetylcysteine; PG—parotid glands; SMG—submandibular glands; COX—cytochrome c oxidase; **** *p* < 0.0001.

An eight-week high-fat diet resulted in a considerable reduction of complex I activity in both parotid (−21%, *p* < 0.0001) and submandibular (−48%, *p* < 0.0001) glands compared to the control group. Chronic treatment with NAC significantly increased the activity of complex I in the parotid and submandibular glands of HFD + NAC rats in relation to the HFD group (+ 42%, *p* < 0.0001; +68%, *p* < 0.0001, respectively), to a level similar to the control rats fed normal chow (Figure 1).

High-fat diet and NAC supplementation did not affect the activity of complex II. In all the studied groups the activity of this complex remained at a similar level as in the control rats fed normal chow (Figure 1).

The activity of complex II + III in the parotid glands of rats fed a high-fat diet, although lower, did not differ from the control group. Chronic treatment with NAC significantly increased the activity of complex II + III in the parotid glands compared to the HFD group (+ 10%, *p* < 0.0001), but its activity in the HFD + NAC group did not differ significantly from the control group.

An eight-week high-fat diet resulted in a significant reduction in the activity of complex II + III in the submandibular glands compared to the control group (−12%, *p* < 0.0001). NAC supplementation prevented the reduction of complex II + III activity on the one hand, because the activity of this complex in the HFD + NAC group did not differ from the control group, but on the other hand it did not differ from the values obtained in HFD group (Figure 1).

COX activity in the parotid (+ 27%, *p* < 0.0001) and submandibular (+ 66%, *p* < 0.0001) glands of rats fed a high-fat diet for eight weeks was significantly elevated compared to the controls. NAC supplementation prevented an increase in COX activity in both salivary glands, as COX activity in the parotid and submandibular glands of HFD + NAC was significantly reduced compared to the HFD group (−40%, *p* < 0.0001, −23%, *p* < 0.0001, respectively) (Figure 1).

### 3.3. ADP/ATP Ratio and H_2_O_2_ Level

**Figure 2:**
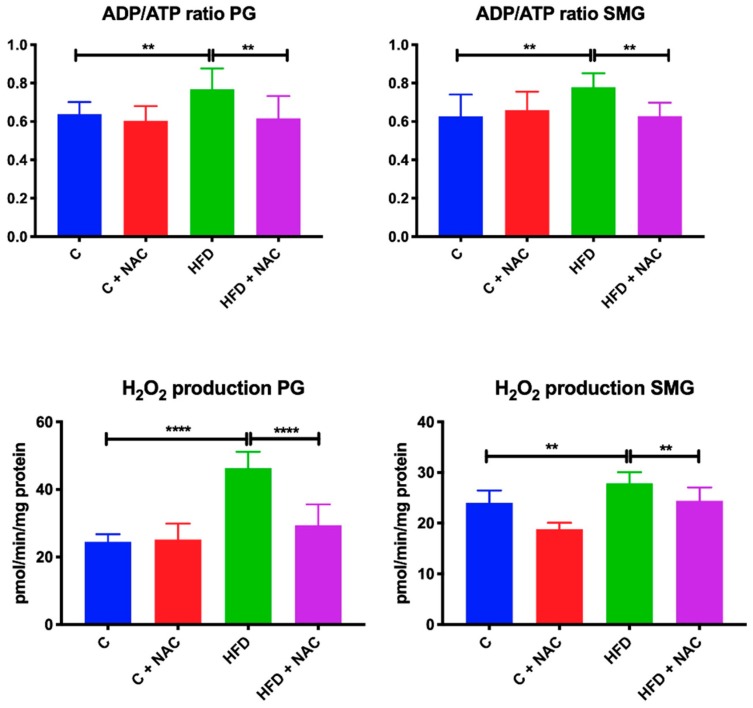
Effect of NAC supplementation on ADP/ATP ratio and hydrogen peroxide production in salivary gland mitochondria. C—control rats; C + NAC—control rats + N-acetylcysteine; HFD—rats fed high-fat diet; HFD + NAC—rats fed high-fat diet + N-acetylcysteine; PG—parotid glands; SMG—submandibular glands; H_2_O_2_—hydrogen peroxide. ** *p* < 0.005, **** *p* < 0.0001.

ADP/ATP ratio in the in the parotid (+ 20%, *p* = 0.0019) and submandibular (+ 24%, *p* = 0.003) glands of rats fed high-fat diet for eight weeks was significantly higher than in the group fed normal chow. NAC supplementation prevented ADP/ATP ratio from increasing in both salivary glands as ADP/ATP ratio in the parotid and submandibular glands of HFD + NAC rats was significantly reduced compared to the HFD group (−20%, *p* = 0.005, −24%, *p* = 0.0034, respectively) (Figure 2).

The level of H_2_O_2_ was significantly higher in the parotid and submandibular glands of rats fed with high-fat diet compared to the control rats (+ 48%, *p* < 0.0001, +14%, *p* = 0.0017, respectively). NAC supplementation prevented the increase in H_2_O_2_ production in both glands, so H_2_O_2_ concentration in the parotid and submandibular glands of HFD + NAC rats was lower compared to the HFD group, and at the same time it did not differ from the control group (−37%, *p* < 0.0001, −14%, *p* = 0.0058, respectively) (Figure 2).

### 3.4. Activity of Mitochondrial CS

**Figure 3:**
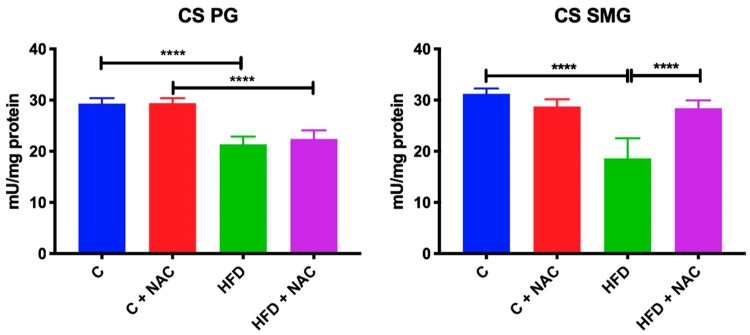
Effect of NAC supplementation on citrate synthase activity in the salivary gland mitochondria. C—control rats; C + NAC—control rats + N-acetylcysteine; HFD—rats fed high-fat diet; HFD + NAC—rats fed high-fat diet + N-acetylcysteine; PG—parotid glands; SMG—submandibular glands; CS—citrate synthase; **** *p* < 0.0001.

An eight-week high-fat diet resulted in a significant reduction of CS activity in both the parotid (−28%, *p* < 0.0001) and submandibular (−39%, *p* < 0.0001) glands compared to the control group. Chronic treatment with NAC considerably increased CS activity in the submandibular glands compared to the HFD group (+ 47%, *p* < 0.0001). In the parotid glands, NAC supplementation prevented the reduction of CS activity on the one hand—because the activity of this complex in the HFD + NAC group did not differ from the controls—but on the other hand CS activity did not differ from the values obtained in the HFD group (Figure 3).

### 3.5. GSH, GSSG and Redox Status

**Figure 4:**
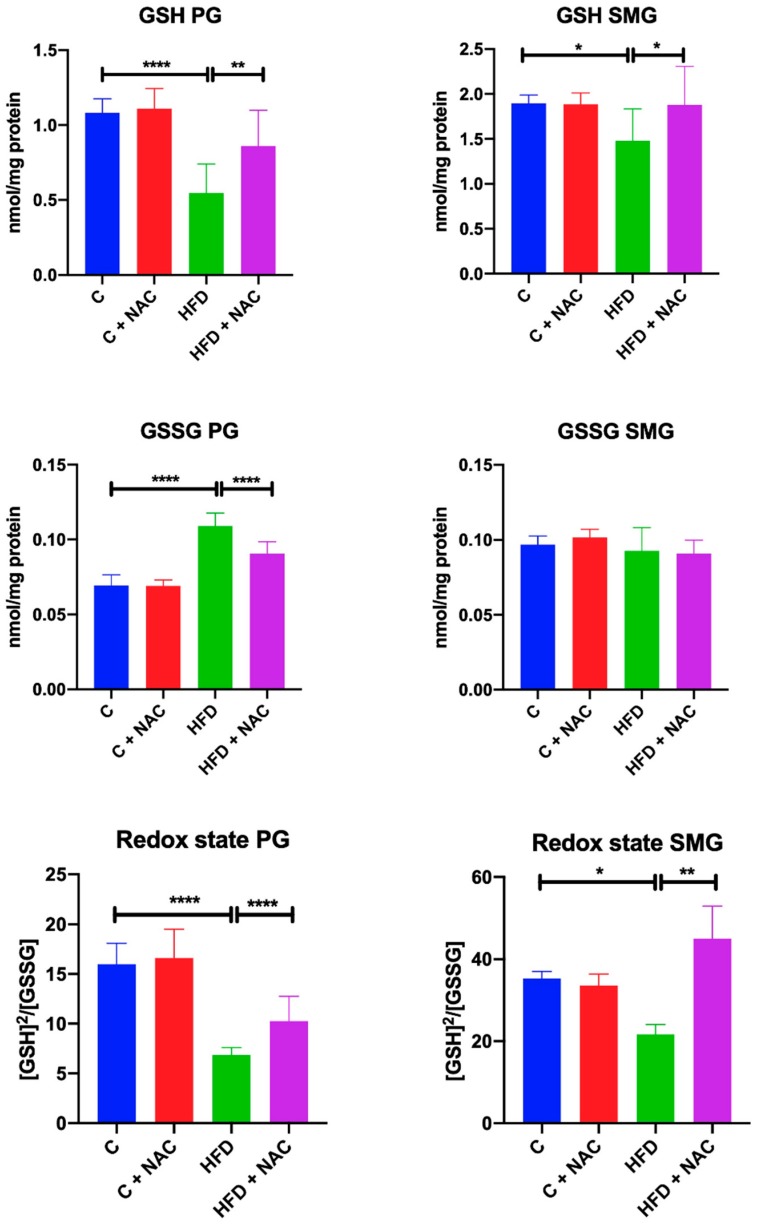
Effect of NAC supplementation on glutathione and redox status in the salivary gland mitochondria. C—control rats; C + NAC—control rats + N-acetylcysteine; HFD—rats fed high-fat diet; HFD + NAC—rats fed high-fat diet + N-acetylcysteine; GSH—reduced glutathione; GSSG—oxidized glutathione; * *p* < 0.05, ** *p* < 0.005, **** *p* < 0.0001.

A high-fat diet consumed for eight weeks resulted in a significant reduction of GSH concentration and redox status in the mitochondria of both parotid (−50%, *p* < 0.0001, −57% *p* < 0.0001, respectively) and submandibular (−21%, *p* = 0.0135, −37%, *p* = 0.04, respectively) glands compared to the control group. In the mitochondria of HFD rats’ parotid glands we also observed a significant increase in GSSG concentration compared to the controls (+ 37%, *p* < 0.0001). NAC supplementation resulted in increased GSH content and redox status in the mitochondria of parotid (+ 56%, *p* = 0.0016, +31%, *p* = 0.0077, respectively) and submandibular (+ 26%, *p* = 0.0194, +51%, *p* = 0.0039, respectively) glands as well as decreased GSSG content (−17%, *p* < 0.0001) in the mitochondria of parotid glands compared to the HFD group and to the GSSG level of the control group (Figure 4).

### 3.6. Activity of NOX and XO

**Figure 5:**
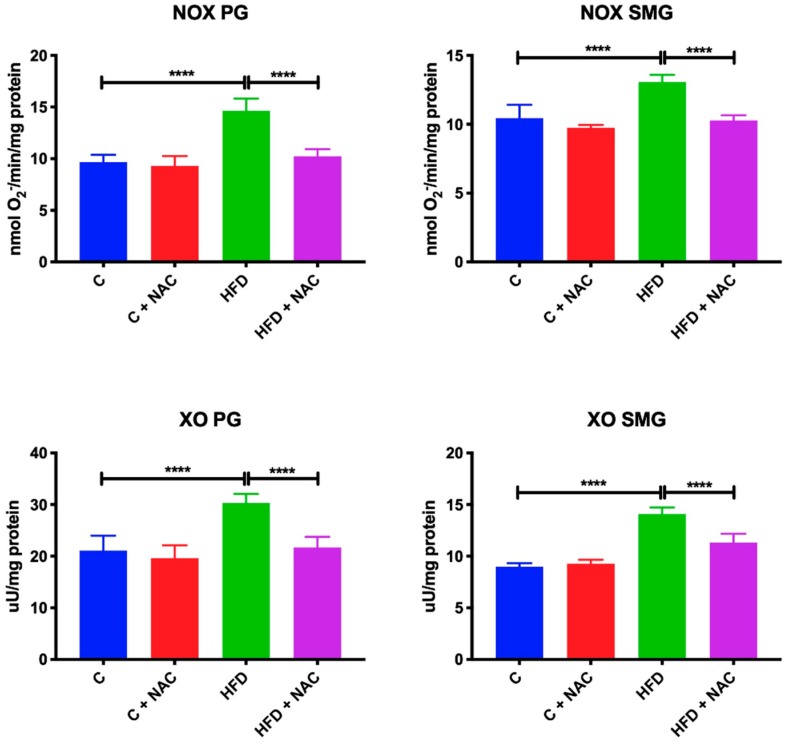
Effect of NAC supplementation on pro-oxidant enzymes in the salivary gland mitochondria. C—control rats; C + NAC—control rats + N-acetylcysteine; HFD—rats fed high-fat diet; HFD + NAC—rats fed high-fat diet + N-acetylcysteine; NOX—NADPH oxidase; XO—xanthine oxidase; **** *p* < 0.0001.

A high-fat diet resulted in increased activity of NOX (+ 54%, *p* < 0.0001, +30%, *p* < 0.0001, respectively) and XO (+ 42%, *p* < 0.0001, +55%, *p* < 0.0001, respectively) in the mitochondria of both parotid and submandibular glands compared to the control group. NAC supplementation prevented an increase in NOX and XO activity in the mitochondria of both salivary glands as the activity of both NOX and XO in parotid (−50%, *p* < 0.0001, −30%, *p* < 0.0001, respectively) and submandibular (−27%, *p* < 0.0001, −22%, *p* < 0.0001, respectively) glands in the HFD + NAC group was significantly lower compared to HFD group (Figure 5).

### 3.7. Inflammation and Apoptosis

**Figure 6:**
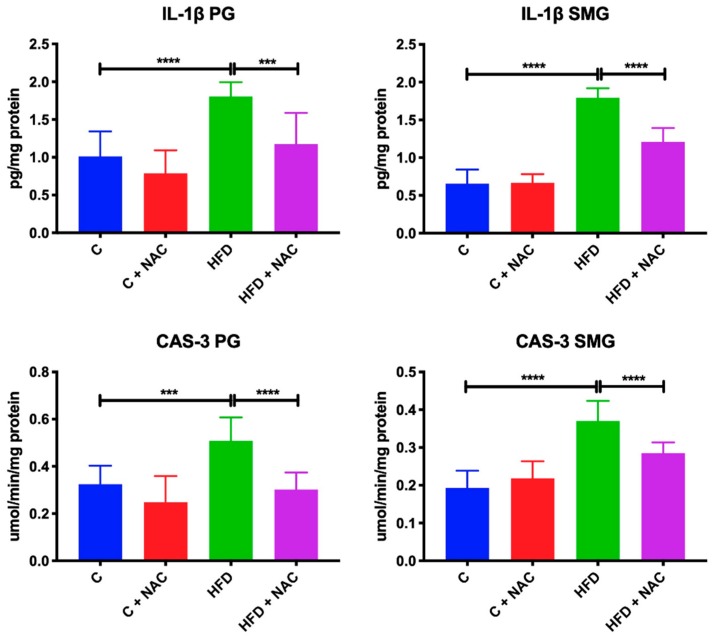
Effect of NAC supplementation on inflammation and apoptosis biomarkers in the salivary gland mitochondria. C—control rats; C + NAC—control rats + N-acetylcysteine; HFD—rats fed high-fat diet; HFD + NAC—rats fed high-fat diet + N-acetylcysteine; IL-1β—interleukin 1β; CAS-3—caspase 3; *** *p* < 0.0005, **** *p* < 0.0001.

HFD rats were characterized by significantly increased IL-1β concentration and CAS-3 activity in the mitochondria of parotid (+ 80%, *p* < 0.0001, +56%, *p* < 0.0001, respectively) and submandibular (+ 172%, *p* < 0.0001; +94%, *p* = 0.0004; respectively) glands compared to the controls. NAC supplementation resulted in decreased IL-1β concentration and CAS-3 activity in the mitochondria of parotid (−33%, *p* = 0.0007; −40%, *p* < 0.0001, respectively) and submandibular (−33%, *p* < 0.0001; −22%, *p* < 0.0001; respectively) glands compared to HFD rats, in relation to the levels observed in the control group (Figure 6).

### 3.8. Nitrosative Stress

**Figure 7:**
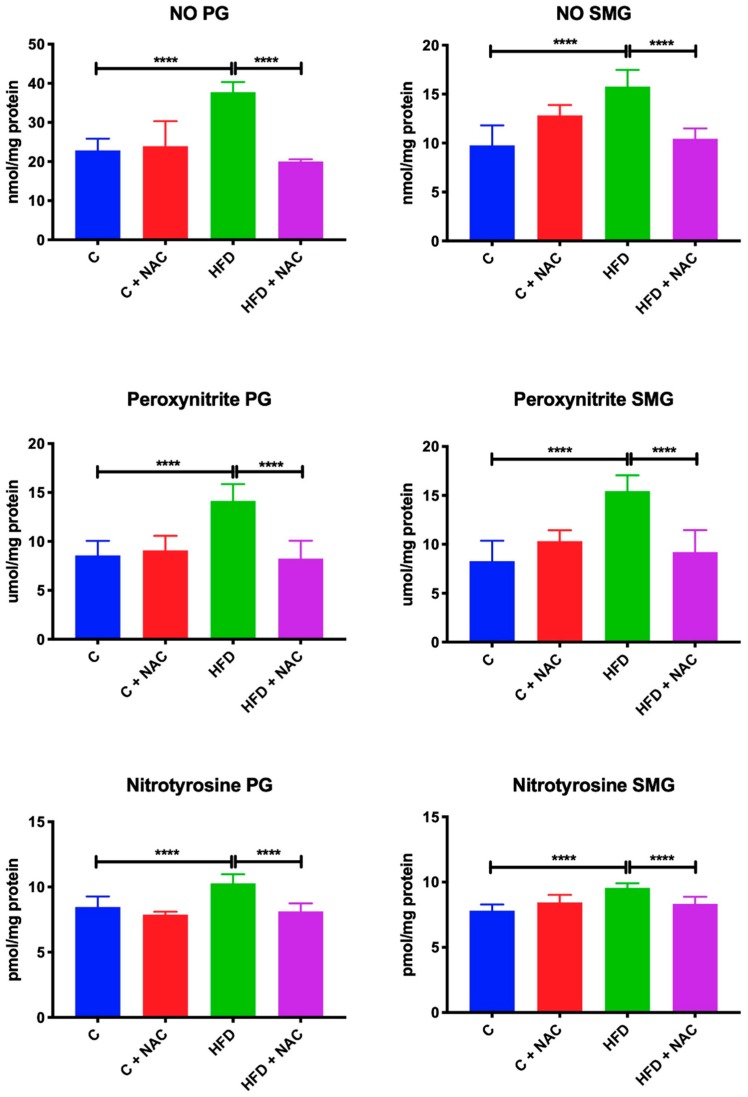
Effect of NAC supplementation on nitrosative stress biomarkers in the salivary gland mitochondria. C—control rats; C + NAC—control rats + N-acetylcysteine; HFD—rats fed high-fat diet; HFD + NAC—rats fed high-fat diet + N-acetylcysteine; NO—nitric oxide; **** *p* < 0.0001.

HFD rats showed significantly increased NO, peroxynitrite and nitrotyrosine concentration in the mitochondria of parotid (+ 65%, *p* < 0.0001; +63%, *p* < 0.0001, +12%, *p* < 0.0001; respectively) and submandibular (+ 62%, *p* < 0.0001; +86%, *p* < 0.0001; +23%, *p* < 0.0001; respectively) glands compared to the controls. NAC supplementation resulted in decreased NO, peroxynitrite, and nitrotyrosine concentration in the mitochondria of parotid (−48%, *p* < 0.0001; −42%, *p* < 0.0001, −19%, *p* < 0.0001; respectively) and submandibular (−50%, *p* < 0.0001; −67%, *p* < 0.0001; −15%, *p* < 0.0001; respectively) glands compared to HFD rats, in relation to the levels observed in the control group (Figure 7).

### 3.9. Correlations

In the parotid and submandibular glands of HFD + NAC rats we observed a negative correlation between GSH concentration and H_2_O_2_ production (r = −0.72, *p* = 0.019; r = −0.902, *p* < 0.0001, respectively) and between GSH and peroxynitrile (r = −0.713, *p* = 0.021; r = −0.801, *p* = 0.005, respectively). In the group of HFD + NAC rats we also demonstrated a positive correlation between CAS-3 activity and IL-2 concentration in submandibular glands (r = 0.936, *p* < 0.0001), and a positive correlation between CAS-3 and NOX activities in parotid glands (r = 0.846, *p* = 0.002).

## 4. Discussion

In our study we assessed the effect of NAC supplementation on salivary glands: Their mitochondrial respiratory system and function, mitochondrial ROS production and glutathione metabolism, the activity of NOX and XO, as well as some parameters of nitrosative stress and apoptosis in a rat model of insulin resistance.

HFD is a suitable model of human IR [13,14,16,42]. As expected, the implemented eight-week high-fat diet resulted in a significant increase in the body weight of rats, and decreased glucose tolerance assessed by a considerably higher concentration of blood glucose and insulin as well as HOMA-IR index compared to the control rats [43,44,45]. It was also observed that HFD induces OS [13,14,15,16] as well as mitochondrial dysfunction and cell death [16] in the salivary glands of rats and mice [30], which was confirmed in our study.

Chronic NAC treatment has been reported to be beneficial in IR and its complications [15,46,47,48]. We confirmed that NAC prevents hyperglycemia and hyperinsulinemia as well as impedes the development of IR. We also confirmed the preventing effect of NAC in increasing body weight seen in HFD rats compared to the HFD + NAC group. Despite the similar calorific value of the consumed food, HFD + NAC rats were characterized by lower final body weight compared to HFD rats, which most probably resulted from NAC-induced reduction in the intestinal absorption of the ingested chow [49]. Żukowski et al. [15] demonstrated that NAC supplementation protects parotid glands against oxidative stress, while the severity and extent of oxidative damage to submandibular glands of HFD + NAC rats was only reduced by NAC treatment. Our results confirmed the observations that NAC prevents parotid gland dysfunction, which was expressed as increased secretion of stimulated saliva in HFD + NAC rats.

The primary source of ROS formation in the body is the respiratory chain in mitochondria. There, electrons are transferred through the mitochondrial electron transport chain (mETC) to reduce molecular oxygen [50,51,52]. Interestingly, the uncontrolled production of ROS in mETC has been implicated in many pathological situations, and has also led to the dysfunction of the salivary glands in many systemic diseases [16,30,53]. Therefore, attempts should be taken to regulate/restore mitochondrial function to prevent oxidative damage to lipids, proteins, and DNA/RNA.

The effect of NAC treatment on mitochondrial: Respiratory complexes, H_2_O_2_ production and GSH, activity of NOX and XO as well as on nitrosative stress, inflammation, and apoptosis in a rat model of insulin resistance has not been described yet. Basically, we proved that NAC treatment provides beneficial effects on mitochondrial function. In fact, parotid and submandibular gland mitochondria of NAC-treated HFD rats showed a significant increase of complex I, II + III, and COX, as well as a decrease of ADP/ATP ratio compared to rats fed a high-fat diet. Thus, NAC reduces H_2_O_2_ production, increases the pool of mitochondrial GSH, and prevents inflammation, apoptosis, and nitrosative stress in the mitochondria of both salivary glands of HFD rats.

Mitochondrial complexes are of great importance as they play a key role in energy production. All respiratory chain enzymes are proteins with active thiol groups that can sense redox status in the cell, and simultaneously make these complexes very sensitive to oxidative modifications. The activity of respiratory chain enzymes is inhibited in the case of redox imbalance of sulfhydryl groups. This balance is disturbed by the deficiency of free sulfhydryl groups that are a ready source of reducing equivalents to quench radical species, which keeps thiol groups of the enzymes in a reduced state also by excess of free radicals [54]. NAC most likely prevents the reduction of mitochondrial complexes activity in HFD rats’ salivary glands by protecting sulfhydryl groups from oxidation, as described for nerve cell mitochondria [55]. Moreover, Banaclocha et al. [9] argue that NAC thiol groups may participate in cofactor and substrate binding by forming covalent addiction products or charge transfer complexes. Thiol groups provided by NAC can also help preserve the tertiary structure of mitochondrial enzymes by serving as a donor for weak hydrogen bonds. The authors believe that these mechanisms can improve the affinity of the enzymes to substrates and the internal electron transfer rate, or change the affinity of the enzymes to oxygen.

Moreover, NAC has an indirect antioxidant effect by providing cysteine and boosting GSH synthesis. The observed increase in GSH concentration may also be related to the previously cited NAC-suppressed NF-ĸB (nuclear factor kappa B) activation and upregulation of the gene expression of this protein [56,57]. It should be noted that mitochondria do not synthesize their own GSH and therefore it must be transported from the cytosol through a multi-component system located in the internal mitochondrial membrane. The observed increase in GSH concentration in the mitochondria of both salivary glands of rats is most probably the result of increased cytosolic GSH concentration and a boosted rate of transport through mitochondrial membranes [58]. The main function of glutathione is to maintain a reduced state of thiol groups of proteins, and thus—similarly to NAC—it can determine efficient functioning of the respiratory chain and oxidative phosphorylation. Another mechanism through which GSH can prevent the dysfunction of respiratory chain enzymes is through direct interaction of this chain with ROS. GSH is used as a cofactor by glutathione peroxidase in the detoxification of H_2_O_2_ and peroxynitrite [59]. Therefore, it is not surprising that with increased GSH concentration we can observe the reduction of H_2_O_2_ and peroxynitrite concentrations in both salivary glands of HFD + NAC rats. It was demonstrated that H_2_O_2_ reacts easily with protein thiol groups as well as peroxynitrite with iron-sulfur cluster of mitochondrial electron transport enzymes, leading to their inactivation [59,60]. Similarly, NAC reacts rapidly with highly reactive oxygen and nitrogen radicals [61]; however, it is considered to be a weak antioxidant [62]. Moreover, the intracellular concentration of NAC, compared to the levels of GSH, ascorbate, and antioxidant enzymes, is too low for NAC to directly affect the antioxidant capacity [63].

It is noteworthy that there were no changes in GSH concentrations in the salivary glands of C + NAC rats, which is consistent with the previous observations [15,64]. It has been demonstrated that thiol supplementation, particularly when GSH content is undisturbed, results in an increased cysteine concentration without a concomitant rise in GSH level [64].

Treatment of rats with NAC resulted in a significant decrease in the ADP/ATP ratio compared to non-treated HFD rats, which was most probably connected with the maintenance of a reduced state of the said sulfhydryl groups of mitochondrial enzymes. It was shown that sulfhydryl groups of these enzymes are essential in the process of oxidative phosphorylation and in energetic metabolism [65]. It should be highlighted that a decreased ADP/ATP ratio can undoubtedly be influenced by the maintained (by NAC supplementation) activity of CS—an enzyme associated with tricarboxylic acid cycle, the efficient functioning of which is enabled by 12 ATP molecules.

Although continual energy production is one of the most important tasks of the mitochondria, these organelles are also involved in the initiation and execution of processes leading to cell death. It has been proven that the reduced activity of ETC (electron transport chain) complexes increases mitochondrial H_2_O_2_ production [66,67]. The observed increase in H_2_O_2_ concentration in the mitochondria of both salivary glands of HFD rats may also be caused by increased NADPH oxidase activity, particularly since mitochondria mainly contain the isoform NOX4 that predominantly produces H_2_O_2_ [68,69]. It should be noted that the increase in NOX activity may result from high concentrations of glucose [70] and free fatty acids [71], all of which were increased in the serum of HFD rats in our study. A certain amount of ROS is also provided by XO which catalyzes one- or two-electron molecular oxygen reduction with the formation of superoxides and H_2_O_2_ [60]. Excessive ROS production helped in inducing inflammation to a large extent. ROS may be implicated in the activation/intensification of numerous signaling pathways such as NF-κB, and cytokine release. Inflammatory cytokines promote cell damage, apoptosis, and consequently, organ damage, which we observed in the mitochondria of salivary glands of rats exposed to a high-fat diet. The observed anti-inflammatory effect of NAC was associated with the decreased activity of NOX and XO as well as lowered IL-2 concentration in both salivary glands of HFD + NAC rats. Further studies are required to explain whether the observed anti-inflammatory effect of NAC supplementation is associated with NAC-induced inhibition of proteasome activity followed by the downregulation of NF-κB activity, or whether this effect could be secondary to the systemic influence of NAC (preventing insulin resistance). The NAC-dependent regulation of mitochondrial ROS production and inflammation appears to be of key importance in reducing the rate of apoptosis in both salivary glands of rats fed a high-fat diet. In our experiment, NAC supplementation resulted in the decreased activity of caspase 3 in both salivary glands, with a simultaneous positive relationship between IL-2 concentration and caspase activity in submandibular glands, and NOX activity and caspase activity in the parotid glands of HFD + NAC rats.

We showed that HFD upregulated NO and peroxynitrite concentrations, as well as enhanced tyrosine nitration of mitochondrial proteins in the salivary glands of rats, which was previously described for the mitochondria of submandibular glands of HFD mice [30]. In the presence of NAC, NO production, and consequently, nitrosative stress were significantly reduced. It should be underlined that by decreasing NO levels in the salivary gland mitochondria, NAC may improve microvascular delivery of oxygen and salivary gland oxygenation and thus prevent changes in saliva secretion. It was demonstrated that hyperbaric oxygen treatment of previously irradiated head and neck cancer patients boosted non-stimulated and stimulated salivary flow rate to a level >0.2 mL/min and >0.7 mL/min, respectively [72].

One of the limitations of our experiment is the lack of histological examination. Thus, we can not show whether the diet in itself and/or NAC administration influence the total mitochondrial content or cause morphological changes in the salivary glands.

## 5. Conclusions

Dietary administration of NAC contributes to the preservation of mitochondrial enzymes in the salivary glands of HFD rats. NAC supplementation enhances energy metabolism in salivary glands of HFD rats by affecting respiratory chain enzymes and citrate synthase. Treatment with NAC prevents inflammation, apoptosis, and nitrosative stress in the salivary glands of HFD rats.

## Figures and Tables

**Table 1:** Effect of N-acetyl-cysteine (NAC) supplementation on body weight, plasma metabolic parameters, food intake, salivary flow rate and salivary gland weight.

	C	C + NAC	HFD	HFD + NAC
Final body weight (g)	280.0 ± 12.31	278.3 ± 26.62	380.9 ± 30.42 *	314.5 ± 32.78 *^,#^
Plasma glucose (mg/dL)	90.67 ± 6.19	91.77 ± 11.84	148.6 ± 7.99 *	98.50 ± 8.41 *^,#^
Plasma insulin (mIU/mL)	78.92 ± 8.46	76.86 ± 9.75	165.4 ± 12.79 *	84.49 ± 17.07 *^,#^
HOMA-IR	2.93 ± 1.36	2.56 ± 2.45	20.11 ± 1.96 *	2.89 ± 1.83 ^#^
Plasma free fatty acids (µmol/L)	75.59 ± 10.99	69.80 ± 8.83	173.4 ± 11.99 *	86.38 ± 12.5 *^,#^
Food intake (mg/day)	20.98 ± 3.95	21.70 ± 6.93	11.12 ± 6.1 *	12.19 ± 5.68 *^,#^
Energy from chow (MJ/day)	0.19 ± 0.1	0.17 ± 0.1	0.31 ± 0.12 *	0.27 ± 0.1 *
Non-stimulated salivary flow (µL/min)	0.4229 ± 0.12	0.3813 ± 0.11	0.3707 ± 0.14	0.4195 ± 0.09
Stimulated salivary flow (µL/min)	110.2 ± 9.77	103.5 ± 10.76	70.08 ± 8.14 *	105.2 ± 12.05 *^,#^

C—control rats; C + NAC—control rats + N-acetylcysteine; HFD—rats fed high-fat diet; HFD + NAC—rats fed high-fat diet + N-acetylcysteine; PG—parotid glands; SMG—submandibular glands; HOMA-IR—homeostasis model assessment of insulin resistance; * *p* < 0.05 vs. control; # *p* < 0.05 vs. HFD.
